# The Effect of Whey Protein Supplements on Acne Vulgaris among Male Adolescents and Young Adults: A Case-Control Study from North of Jordan

**DOI:** 10.1155/2024/2158229

**Published:** 2024-04-09

**Authors:** Jihan Muhaidat, Almutazballlah Qablan, Faris Gharaibeh, Ghaith H. Albataineh, Nour Abdo, Diala Alshiyab, Firas Al-Qarqaz

**Affiliations:** ^1^Department of Dermatology, Faculty of Medicine, Jordan University of Science and Technology, P.O. Box 3030, Irbid 2110, Jordan; ^2^Faculty of Medicine, Jordan University of Science and Technology, P.O. Box 3030, Irbid 2110, Jordan; ^3^Department of Public Health and Family Medicine, Faculty of Medicine, Jordan University of Science and Technology, P.O. Box 3030, Irbid 2110, Jordan

## Abstract

**Background:**

Young people and athletes willing to gain muscle mass and strength are likely to consume whey protein supplements. The effect of milk as a dietary source of whey protein on acne is still controversial. At the same time, a few studies have suggested an acnegenic impact of whey protein supplements.

**Objectives:**

To examine the association of whey protein supplements on acne risk among male adolescents and young adults.

**Materials and Methods:**

201 male teenagers and young adults attending fitness centers in Irbid/Jordan were involved in an observational case-control research; those with acne were deemed cases, and those without acne were considered controls. The primary outcome was a comparison of the proportion of participants in each group who consumed whey protein supplements within the previous three months.

**Results:**

100 acne-afflicted participants were compared to 101 healthy controls with similar demographics, including age, body mass index, educational level, and smoking habits, as well as intake of vitamin B12, corticosteroids, and anabolic steroids. However, considerably more participants in the acne group (47%) were taking whey protein supplements than in the control group (27.7%) (*p*=0.0047). The significance of this difference was maintained after multivariate analysis.

**Conclusion:**

This case-control study provides evidence of a positive association between whey protein consumption and acne risk.

## 1. Introduction

Acne is a disease of the pilosebaceous unit that is triggered by androgens. Acne lesions are caused by a combination of four primary pathogenic factors: increased and altered sebum production under the control of androgens (increased sensitivity of the androgens receptors), abnormal keratinization leading to comedones, the proliferation of *Propionibacterium acnes*, and release of inflammatory mediators into the skin [1, 2].

Acne is a complex condition of the pilosebaceous unit that may be exacerbated or alleviated by various factors, including the diet one maintains. The carbohydrate-rich diet stimulates androgen release by increasing the secretion of insulin and serum insulin growth factor-1 (IGF-1) and stimulates forkhead box protein O1 (FoxO1)/mammalian target of rapamycin complex 1 kinase (mTORC1) pathway, leading to the expression of cytokines, follicular hyperkeratinisation, and increased sebum production [3]. Milk and dairy products containing whey and casein protein increase the plasma levels of postprandial insulin and IGF-1, especially in the Western diet [4].

Although milk has a low glycemic index, it can aggravate acne by increasing insulin and insulin growth factor-1 (IGF-1) levels and releasing comedogenic hormones like estrogen, progesterone, androgen precursors, and 5*α*-reductase steroids [5, 6].

Casein and whey proteins are the main proteins of milk. Casein makes up about 80% (29.5 g/L) of the total protein in cow's milk, and whey protein makes up about 20% (6.3 g/L) [7]. Whey protein is the liquid left over after milk has been curdled and filtered to remove casein (curds). To convert whey into protein powder, whey is filtered and dried in multiple steps. It is a mixture of globular proteins that are water-soluble by-products of cheese production, containing five primary proteins: *β*-lactoglobulin, *α*-lactalbumin, glycomacropeptide, protease peptone 3, immunoglobulins, and serum albumin, which account for approximately 85% of whey protein [8, 9].

Whey protein supplements are trendy among athletes and bodybuilders. They positively affect muscle size, strength, and athletic performance without significant adverse effects. Additionally, whey is rich in bioactive compounds that may reduce disease risk [8]. However, some adverse effects on the kidney and liver function are potential, especially if consumption is chronic or without professional guidance [10].

A few prior studies based on case series and uncontrolled observational studies discovered a correlation between whey protein supplements and acne, particularly in athletes who took whey protein. This study aims to investigate the association between acne and the consumption of nutritional supplements, such as whey protein, among Jordanian athletes and young adults who attend fitness centers.

## 2. Materials and Methods

### 2.1. Study Design

This observational case-control study was conducted from June to September 2022. Fitness center attendees who participated in the study were interviewed to fill out an anonymous questionnaire about their demographics and intake of supplements during the previous three months. They were then examined for the presence of acne. Participants with acne were considered the cases, and those without evidence of acne were the controls. An equal number of cases and controls were included; all were from one gender and age-matched. The ethics committee of Jordan University of Science and Technology approved the study (IRB number 2022/379). Participants and guardians of those under 18 years old verbally consented to participate in the study.

### 2.2. Study Population and Sample

Protein supplement consumption is common among athletes and young people who seek enhancement of their muscle mass or strength, so our sample was recruited from multiple fitness center attendees in Irbid/Jordan. We enrolled 201 males between 14 and 43 years old, 100 cases of acne versus 101 healthy controls.

### 2.3. Measurements

In this study, a three-section questionnaire was used to collect data. The questionnaire was filled out by researchers using the face-to-face interview method. The first section was about the participants' characteristics, including age, educational level, body mass index (BMI), smoking habits, chronic diseases, family history of acne, and factors predisposing to acne, including corticosteroid, vitamin B12, and anabolic steroid intake in the last three months. The participants in the second section discussed the use of non-dietary protein supplements (whey, casein, and creatine). The duration of use in weeks and the frequency of intake per week were also recorded, and the consumption of full-fat milk, skimmed milk, and dairy products was also evaluated. Two servings or more were regarded as significant daily consumption. All participants were then examined for acne; those with acne or who had been on acne treatment were considered cases. The third section was completed for 100 acne patients, including the duration, site, and type of acne lesions. The severity of acne was then calculated using the Global Acne Grading System score as follows: (1–18) mild, (19–30) moderate, (31–38) severe, and (≥39) very severe [11]; however, we considered (≥31) as severe because of the shortage of participants in the very severe category. The primary outcome measures were comparing the percentage of participants on different protein supplements, milk, and dairy products. The secondary outcome was to study the correlation between acne severity, demographics, and supplement intake.

### 2.4. Statistical Methods

Statistical analysis was performed using SAS 9.4 (Cary, NC). Continuous variables are presented as mean and standard deviation. Frequency and percentage values were used to present categorical (qualitative) variables. Descriptive analysis was performed to elicit the percentage, mean, and standard deviation (SD) of the quantitative data. The chi-square test was used to compare acne and control group values for categorical variables, including participants' characteristics and non-dietary supplement intake. Differences are considered statistically significant if the *p* value < 0.05 and highly significant if the *p* value < 0.01. Bivariate significant results were entered into multivariate logistic regression.

## 3. Results

### 3.1. Characteristics of the Participants

The study included 201 male participants, 100 acne vulgaris patients, and 101 acne-free controls; their ages ranged from 14 to 43 years. The mean age of the acne group was (22 ± 4.52) years, and the mean age of the control group was (23 ± 5.07) years. The age was also comparable when divided into intervals without significant differences between the two groups. The two groups were also similar regarding educational level, body mass index, smoking, and chronic diseases (hypertension, diabetes mellitus, and thyroid disease). The intake of corticosteroid, vitamin B12, and anabolic steroids in the last three months was not different between cases and controls. On the other hand, there was a significant difference between the acne and control groups in terms of family history of acne (*p*=0.0142); these results are shown in Table 1.

### 3.2. High Intake of Dairy Products

High intake, which was defined in this study as the consumption of two or more servings of dairy products, was also compared between the two groups. This includes full-fat, skimmed, and other dairy products (cheese and yogurt). 40% of acne patients and around 30% of controls consumed high amounts of full-fat milk; this difference was significant (*p*=0.0356), while the consumption of both skim milk and other dairy product (yogurt and cheese) was not statistically significant between cases and controls.

### 3.3. Protein Supplement Intake

Around half of all participants in our study were on non-dietary protein supplements, 59% of acne patients versus 42.5% of controls; this difference was statically significant (*p*=0.0199); this finding is shown in Table 1. Further detailing the type of protein supplements, whey protein supplements were the most popular supplement, with approximately 74% of protein supplement users consuming them; whey supplements were used by 47 percent of acne patients and 27.7 percent of the control group. This difference was statistically significant (*p*=0.0047). The intake of other protein supplement types (casein, whey, and creatine) did not differ significantly between the two groups. The duration of protein supplement usage ranged from one week to three years, with the majority of users using supplements for more than three months. There was no difference in protein supplement use duration between cases and controls. In addition, over half of the cases and controls took protein supplements daily, and their weekly consumption was comparable. These results are displayed in Table 2.

### 3.4. Acne Assessment and Factors Affecting the Severity

The face was the most common site of acne in 80 percent of patients, followed by the back in 43 percent, the shoulders in 28 percent, and the chest in 11 percent. The severity of acne was mild in 66% of cases (GAGS 1–18), moderate in 19% of cases (GAGS 19–35), and severe in only 15% of cases (GAGS >31). Our acne patients showed no correlation between acne severity and age, BMI, smoking status, vitamin B12, corticosteroids, or protein supplements.

### 3.5. Multivariate Analysis of Results

Multivariate logistic regression was done for the significant bivariate results; the variables included family history, protein supplement intake, whey protein, and full-fat milk, and this yielded the following results. The odds of having acne were 2.94 (around three times) for participants who consume whey protein compared to controls (OR: 2.94, CI: 1.11–7.82, *p*=0.03). In contrast, the difference between acne patients and controls in terms of family history, intake of full-fat milk, and protein supplementation became insignificant.  Table 3 illustrates these findings.

## 4. Discussion

This study aimed to investigate the relationship between acne vulgaris and protein supplements. This is especially important for athletes and young adults who regularly consume concentrated protein preparations to increase muscle strength and mass. This age group is also associated with a higher risk of acne. So, it is unclear what effect these factors have on acne causation or aggravation.

We studied an equal number of acne patients and healthy controls whose demographics and consumption of known acne triggers were matched. A more significant proportion of acne patients used whey supplements than control subjects. Chi-square analysis revealed that family history and consumption of full-fat milk differed significantly; however, multivariate analysis rendered the difference insignificant. Consequently, our findings imply that whey supplements affect acne formation.

Few previous studies, based on case series and small patient populations, have suggested the acnegenic effect of whey protein supplements [12–15]. Our results supported this potential effect. A recent clinical trial involving a small number of acne patients demonstrated no inferior effect of whey protein supplements on acne count or severity [16]. In contrast to previous research, our study included a significantly greater number of acne patients along with controls of the same gender and age range.  Table 4 summarises the published studies of the effect of whey protein supplements on acne vulgaris.

In this study, the percentages of participants from both groups who consumed whey from dietary sources, specifically skim milk, cheese, and yogurt, were very similar. Although a more significant proportion of acne sufferers reported drinking full-fat milk, this difference lost statistical significance when other factors were considered in the study.

Several studies have demonstrated that milk consumption leads to an increased risk of acne [5, 17, 18]. In girls, total milk intake, low-fat milk intake, and skim milk intake were associated with acne, but in boys, only skim milk was associated with acne. Another 2489-person prospective cohort study linked acne to high dairy consumption. Recent case-control studies have revealed that the frequency of milk consumption is higher among acne patients than controls [19, 20]. In contrast, many studies have found no correlation between milk consumption and acne [21, 22]. The potential association between milk consumption and acne risk remains contentious. In addition, it is unknown whether the acne-aggravating impact of milk is related to its many types, such as full-fat, low-fat, and skim milk. Or whether the frequency of milk consumption significantly affects acne development and whether milk impacts acne severity. A more recent meta-analysis to answer these queries found that milk consumption was associated with an increased risk of acne and recommended limiting milk consumption for prevention [23].

In milk and its derivatives, insulin-like growth factor 1 (IGF-1) is present, a molecule related to the stimulation of the growth and division of epidermal cells, the creation of sebum, and the elevation of estrogen factors, all of which contribute to the development of acne [4, 5]. Thus, this may be one of the reasons why a high intake of whey protein products is associated with the development of acne lesions.

Milk products differ from other carbohydrate-containing diets in that they induce significant insulin responses despite their low glycemic index (GI). Milk's insulinotropic mechanism is unknown; several studies have found that reconstituted milk powder and whey have significantly lower postprandial glucose, implying that whey protein is most insulinotropic and may improve postprandial blood glucose levels [24, 25].

## 5. Conclusions

In conclusion, our findings add to the evidence of whey protein's association with acne vulgaris, most likely due to its insulinotropic effect. These findings assist athletes, dermatologists, and general physicians in understanding the development of acne after the initiation of whey supplements and deciding to stop or encourage their discontinuation, particularly in cases of treatment failure or resistance. Considering that our study has limitations due to its cross-sectional design and the small number of patients, the results must be validated by future prospective studies on a larger number of patients, combined with the measurement of insulin and insulin growth factor-1 (IGF-1) levels.

## Figures and Tables

**Table 1:** Comparison of demographics, acne-inducing factors, and dairy and protein supplement consumption between acne cases and controls.

Variable	Cases *N* = 100		Controls *N* = 101		*p* value
	*n*	(n/N)%	*n*	(n/N)%	
Age (years)					0.105
Mean (SD)	22 (4.52)		23 (5.07)		
14–21	49	(49.0)	42	(41.6)	0.168
22–29	43	(43.0)	41	(40.6)	
30–37	7	(7.0)	13	(12.9)	
38–45	1	(1.0)	5	(4.9)	
Education level					0.271
Primary school	15	(15.0)	14	(13.9)	
High school	18	(18.0)	21	(20.8)	
Bachelor	64	(64.0)	58	(57.4)	
Grand	3	(3.0)	8	(7.9)	
Body mass index (BMI)					0.144
Underweight <20	7	(7.0)	4	(4.0)	
Normal weight (20–25)	46	(46.0)	40	(39.6)	
Overweight >25	40	(40.0)	40	(39.6)	
Obese >30	7	(7.0)	17	(16.8)	
Smoking habits					0.850
I don't smoke	39	(39.0)	41	(40.6)	
I smoke cigarettes and water pipe	11	(11.0)	13	(12.9)	
Cigarette smoking	34	(34.0)	35	(34.6)	
Water pipe smoking	16	(16.0)	12	(11.9)	
Chronic disease (hypertension, diabetes mellitus, and thyroid disease)	5	(5.0)	1	(1.0)	0.118^*∗*^
Corticosteroid intake in the last 3 months	4	(4.0)	3	(2.97)	0.690
Vitamin B12 intake in the last 3 months	33	(33.0)	24	(23.8)	0.146
Family history of acne	41	(41.0)	25	(24.8)	0.014
Anabolic steroid intake	2	(2.0)	2	(1.98)	1.000^*∗*^
Protein supplement intake	59	(59.0)	43	(42.5)	0.019
Full-fat milk	44	(44.0)	30	(29.7)	0.035
Skimmed milk	25	(25.0)	31	(30.7)	0.368
Dairy products (yogurt and cheese)	60	(60.0)	59	(58.4)	0.819

^*∗*^These *p* values were obtained using Fisher's exact test due to low count.

**Table 2:** Comparison of the type, duration, and weekly frequency of protein supplement consumption between cases and controls.

Variable	Cases *N* = 59		Controls *N* = 43		*p* value
	*n*	(n/N)%	*n*	(n/N)%	
Whey supplement	47	(79.6)	28	(65.1)	0.004
Casein and whey supplements	8	(13.5)	9	(20.9)	0.982
Creatine supplement	4	(6.7)	6	(13.9)	0.301
Duration of protein supplements (weeks)					0.099
1–4	14	(23.7)	16	(37.3)	
5–8	11	(18.6)	2	(4.6)	
9–12	9	(15.3)	4	(9.3)	
>12	25	(42.4)	21	(48.8)	
Frequency of supplement intake (days/week)					0.284
3	2	(3.4)	2	(4.7)	
4	7	(11.9)	2	(4.7)	
5	12	(20.3)	14	(32.5)	
6	10	(16.9)	5	(11.6)	
7	28	(47.5)	20	(46.5)	

**Table 3:** Multivariate analysis of results.

Variable	Odds ratio	95% Wald confidence limits	*p* value
Family history of acne	2.11	0.03–1.28	0.137
Full-fat milk	1.57	0.82–3.00	0.176
Skimmed milk	0.75	0.23–1.07	0.323
Dairy products (yogurt and cheese)	1.06	0.12–2.17	0.733
Protein supplement intake	0.83	0.33–2.09	0.687
Whey supplement	2.94	1.11–7.82	0.030
Casein and whey supplement	1.70	0.24–2.02	0.097
Creatine supplement	1.81	0.07–1.03	0.448

**Table 4:** Studies on whey supplements and acne.

Reference	Type of study	Patients		Finding
		Number	Gender	
Silverberg [13]	Case series	5	Male	Male patients aged 14 to 18 who developed acne quickly after starting whey protein intake

Simonart [12]	Case series	5	Male	Male adult patients who consumed whey protein developed acne

Pontes et al. [14]	Prospective observational non-controlled	11	Female	Thirty patients on protein supplements were examined
		19	Male	Acneiform lesions developed, females, acne-free people, and those without a history of acne were more affected

Cengiz et al. [15]	Case series	6	Male	Male adolescent patients developed acne on their trunks after consuming whey protein supplements for rapid bodybuilding

Sompochpruetikul et al. [16]	Double-blind randomized controlled trial	25 versus 24	Male	No difference in acne count or severity between 25 acne patients on whey supplements versus 24 acne patients on non-whey protein supplements

## Data Availability

The current study's datasets are available upon reasonable request from the corresponding author.
